# Intravitreal ziv-aflibercept for myopic macular neovascularization after pars plana vitrectomy and silicone oil tamponade: a case report

**DOI:** 10.1097/MS9.0000000000003919

**Published:** 2025-09-15

**Authors:** Sedra Abu Ghedda, Rashed Aljundi, Marwa Baba, Ameen Marashi

**Affiliations:** a Faculty of Medicine, University of Aleppo, Aleppo, Syria; b Marashi Eye Clinic, Aleppo, Syria; c Marashi Eye Clinic, Aleppo, Syria

**Keywords:** macular neovascularization, myopia, silicone oil, vitrectomy, ziv-aflibercept

## Abstract

**Introduction and importance:**

Myopic macular neovascularization (mMNV) is a significant complication of pathological myopia, leading to vision impairment. The primary treatment involves anti-VEGF therapy, with ziv-aflibercept emerging as a cost-effective alternative to traditional therapies. However, the presence of silicone oil in the vitreous cavity, often used in complex vitreoretinal surgeries, poses challenges for drug delivery. This case report aims to explore the efficacy of intravitreal ziv-aflibercept in treating mMNV in a silicone oil-filled eye.

**Case presentation:**

A 66-year-old woman with a history of high myopia underwent vitrectomy and silicone oil tamponade. She presented with significantly reduced visual acuity. Spectral-domain optical coherence tomography (SD-OCT) imaging revealed mMNV, subretinal fluid, and signs of hypotony in her right eye. Given these findings, the patient was administered an intravitreal injection of ziv-aflibercept.

**Clinical discussion:**

Post-injection, the patient exhibited marked improvements, including enhanced visual acuity improving to counting fingers at a distance of 2 m and decreased intraocular pressure to 6 mmHg. SD-OCT imaging demonstrated resolution of subretinal fluid, indicating successful treatment of mMNV. These positive outcomes were maintained over a follow-up period of 6 months, suggesting that ziv-aflibercept can effectively manage mMNV even in the presence of silicone oil.

**Conclusion:**

Intravitreal ziv-aflibercept injection is a viable early treatment option for patients with mMNV in silicone oil-filled eyes. This case highlights its potential to achieve mMNV regression and improve visual acuity, thereby preventing irreversible vision loss in affected individuals. Further studies are warranted to establish standardized protocols for its use in similar clinical scenarios.

## Introduction

Myopic macular neovascularization (mMNV) is a significant vision-threatening complication that occurs in approximately 5-11% of individuals with pathological myopia[1]. This condition is believed to stem from the excessive elongation of the ocular globe, leading to mechanical stress, retinal damage, and dysregulation of proangiogenic and antiangiogenic factors, resulting in mMNV development[2].

Anti-VEGF therapy is the primary treatment approach for mMNV. Ranibizumab is the sole FDA-approved anti-VEGF agent for this purpose, although bevacizumab is frequently used off-label^[2,3]^. Recently, ziv-aflibercept, which was initially utilized in oncology cases (FDA approval since 2012), has been found to be a new avenue for addressing ophthalmic concerns, particularly in regions where the ophthalmic form aflibercept (Eylea) remains financially out of reach. Using ziv-aflibercept as an alternative to aflibercept for specific retinal diseases is under investigation, regardless of its safety concerns related to differences in osmolarity and possible effects on retinal function and structure. Aflibercept is isosmolar compared to the human vitreous and undergoes a different purification process, while ziv-aflibercept is hyperosmolar compared to the human vitreous. Although these two drugs are identical in structure, they are produced through different purification processes and buffer solutions and that may affect their effectiveness and safety in treating retinal disorders. This adaptation of ziv-aflibercept, known as Zaltrap, is emerging as a potential cost-effective alternative because it is effective at managing active mMNV, although further studies are needed to validate its clinical utility[3].

**HIGHLIGHTS**

- The presence of silicone oil in the eye can complicate the treatment of myopic macular neovascularization (mMNV), making effective management more challenging.
- Administering ziv-aflibercept in eyes filled with silicone oil may serve as a viable treatment option for mMNV, potentially leading to improved outcomes.
- Ziv-aflibercept represents a valuable alternative to other anti-VEGF therapies, particularly in third-world countries, where it may offer a more accessible and affordable solution for treating mMNV.

Silicone oil serves as a crucial adjunct in managing complex vitreoretinal surgeries, particularly in cases where conventional approaches may yield poor outcomes[4]. However, its presence can affect the delivery and concentration of drugs injected into the posterior segment of the eye[5]. The occurrence of MNV in eyes filled with silicone oil is considered rare, presenting an opportunity for exploring intravitreal anti-VEGF therapy as a treatment option in oil-filled eyes^[6,7]^. Limited studies exist on the outcomes of anti-VEGF therapy, including bevacizumab and ranibizumab, for mMNV in silicone oil-filled eyes, but these studies have shown promising results^[6,7]^. In this context, we report a successful case of intravitreal ziv-aflibercept treatment for myopic MNV in the presence of silicone oil tamponade. This case report highlights the potential of intravitreal ziv-aflibercept as a treatment option for mMNV in eyes filled with silicone oil. This situation is rarely documented in the literature, as the general practice is to remove silicone oil prior to initiating treatment.

## Case presentation

A 66-year-old Arab woman with a history of high myopia presented following pars plana vitrectomy with silicone oil tamponade for endophthalmitis in her right eye. After surgery, her best corrected visual acuity (BCVA) in the right eye was limited to hand motion, while her left eye exhibited a BCVA of counting fingers with a refraction of −0.50–2.50 × 110. Upon thorough ophthalmic examination, the patient was found to be aphakic with silicone oil in the right eye, along with evident subretinal hemorrhage and myopic degeneration. Conversely, her left eye displayed pseudophakia with myopic degeneration and showed an old chorioretinal scar upon fundus examination.

**Figure 1.**
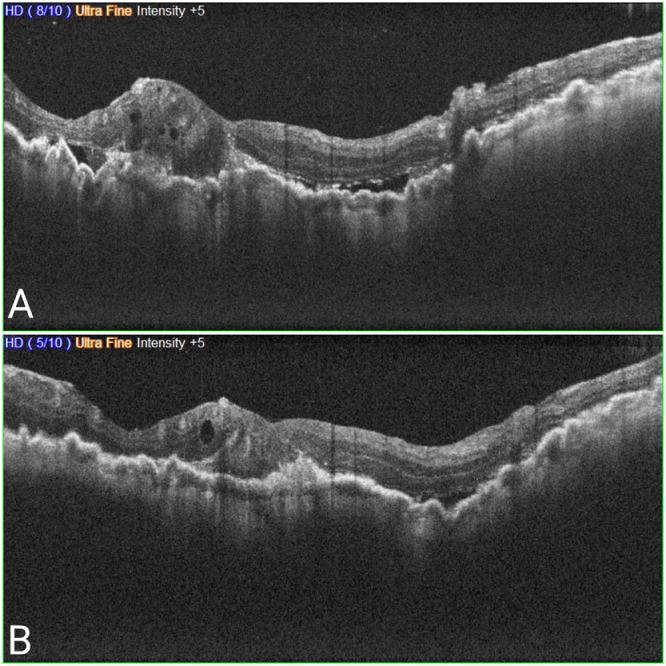
(A) OCT of the right eye prior to Ziv-aflibercept injection demonstrating choroidal folds accompanied by active MNV and the presence of subretinal fluid alongside intraretinal cystic alterations. (B) Subsequent OCT imaging of the right eye following a month of a single administration of Ziv-aflibercept revealed resolution of both subretinal and intraretinal fluid, with only minimal residual intra- and subretinal fluid observed.

Intraocular pressure measurements revealed a substantial discrepancy between the eyes, with the right eye registering at 3 mmHg and the left eye at 11 mmHg. Subsequent spectral-domain optical coherence tomography (SD-OCT) imaging revealed MNV with accompanying subretinal fluid and features of hypotony in the right eye (Fig. 1). The left eye exhibited a distinct hyperreflective lesion reminiscent of a chorioretinal scar. The patient suffers from anxiety and a high vasovagal response, which was observed during her fundus exam and resulted in multiple episodes of vomiting; thus, the decision was made to administer the injection without removing the silicone oil to prevent the patient more irritating experiences.

Given the clinical findings, a therapeutic strategy involving intravitreal ziv-aflibercept was deemed appropriate. One intravitreal injection of 0.05 ml of 1.25 mg of ziv-aflibercept (Zaltrap) was administered. Following the injection, the patient underwent regular follow-up assessments. One month post-injection, a notable improvement in the patient’s BCVA was observed, with her vision improving to counting fingers at a distance of 2 m. Additionally, her intraocular pressure improved to 6 mmHg with no signs of clinical toxicity. Moreover, OCT imaging revealed a significant resolution of subretinal fluid, indicating a favorable response to treatment (Fig. 1). However, FA imaging was not available due to her vomiting issues. During a follow-up period of 6 months, the patient experienced no complications or relapse, maintaining consistent visual acuity.

## Discussion

Macular neovascularization (MNV) can result in vision loss in eyes filled with silicone oil, although its occurrence is uncommon in such cases, leading to limited research on its treatment[6]. Anti-VEGF therapies such as ranibizumab and bevacizumab are typically the primary approach for managing mMNV[2]. Recently, a study proposed Ziv-aflibercept as a promising and safe option for active mMNV; however, larger studies are needed to validate its efficacy[3]. In our case, we administered a 0.05 ml/1.25 mg injection of Ziv-aflibercept for treating mMNV.

Treating mMNV in silicone oil-filled eyes is challenging due to the lack of comprehensive studies on the outcomes of intravitreal anti-VEGF therapy in such patients (Table 1). Concerns include the diffusion of anti-VEGF agents out of silicone oil and their accumulation underneath the oil by gravity, potentially leading to clinical toxicity[5]. Our patient did not exhibit signs of toxicity or accumulation.

**Table 1:** Summary of findings on anti-VEGF injections in eyes with silicone oil

Study	Study type	Condition	Treatment	Visual acuity improvement	Complications	Follow-up duration
Our study	Case report	mMNV	Ziv-aflibercept	Hand motion to counting fingers	None	6 months
Cascavilla *et al* (2013)	Case report	mMNV	Ranibizumab	Counting fingers to 20/100	None	1 year
Xu *et al* (2012)	Case report	mMNV	Ranibizumab	20/200 to 20/160	None	1 month
Salman *et al* (2013)	Case series	INV	Bevacizumab	LogMAR 1.25 ± 0.2 to 0.97 ± 0.3	IOP elevation and Subconjunctival hemorrhage	6 months
Falavarjani *et al* (2010)	Case series	NVG	Bevacizumab	LogMAR 1.7 ± 0.2 to 1.5 ± 0.3	IOP elevation	3 months

A study by Xu et al. on rabbits to determine the effects of silicon oil on the concentration and pharmacokinetics of bevacizumab indicated a reduced concentration and shorter half-life compared to those of normal eyes, suggesting potential treatment inefficacy in the presence of oil^[7,8]^.

Despite these challenges, a few studies have reported successful outcomes with intravitreal anti-VEGF therapy in silicone oil-filled eyes. For instance, in one case reported by Cascavilla *et al*, one patient with mMNV received a ranibizumab injection (0.05 ml/0.5 mg) which led to vision acuity improvement from counting fingers to 20/100 after 2 months without complications or the need for oil removal[7]. Similarly, in another study with mMNV in a silicone oil-filled eye, visual acuity improved from 20/200 to 20/160 a month following intravitreal ranibizumab injection, although subsequent oil removal and intravitreal bevacizumab were necessary afterward[6].

Our study also demonstrated visual acuity improvement from hand motion to counting fingers at a distance of 0.5 m without complications or the need for silicone oil removal after ziv-aflibercept injection. This finding underscores the potential effectiveness of this treatment modality, especially when it is initiated promptly upon the onset of active MNV.

Other studies have explored the use of intravitreal anti-VEGF therapy in silicone oil-filled eyes for conditions other than mMNV, such as iris neovascularization (INV) and neovascular glaucoma (NVG), after diabetic retinopathy.

One study assessed the efficacy of injecting intravitreal bevacizumab into oil-filled eyes with INV caused by diabetic retinopathy. Bevacizumab was injected into silicone oil in fifteen eyes with INV, resulting in regression of INV in all patients and improved visual acuity in twelve eyes. Four patients experienced INV recurrence within 10 weeks and required reinjection[9]. Another study by Falavarjani *et al* on NVG after diabetic retinopathy revealed regression of NVG in five silicon-filled eyes injected with bevacizumab, with one experiencing recurrence and requiring reinjection[5]. The patient in our case experienced no recurrence or complications during a 3-month follow-up period.

Our findings contribute to the growing body of evidence supporting the efficacy of ziv-aflibercept in treating neovascular eye conditions, particularly mMNV. However, it is important to note that this is only a case report, which limits the generalizability of our conclusions. Therefore, further prospective studies with larger sample sizes are necessary to validate our findings.

Additionally, this case represents the first assessment of ziv-aflibercept usage in silicone oil-filled eyes, which yielded positive outcomes and averted complete visual loss.

## Conclusion

In conclusion, our study suggests that ziv-aflibercept injection may be a treatment option for patients with mMNV in silicone oil-filled eyes, with potential benefits in terms of MNV regression and visual acuity improvement. We advocate for its early administration upon the detection of active MNV to mitigate the risk of irreversible vision loss. This conclusion is constrained by the single-case nature of the study, highlighting the crucial need for larger-scale investigations to provide more comprehensive and reliable findings.

## Data Availability

All the data generated or analysed during this study are included in this published article.
